# Preferences and Experiences of People with Chronic Illness in Using Different Sources of Health Information: Results of a Mixed-Methods Study

**DOI:** 10.3390/ijerph182413185

**Published:** 2021-12-14

**Authors:** Svea Gille, Lennert Griese, Doris Schaeffer

**Affiliations:** 1 Interdisciplinary Centre for Health Literacy Research, School of Public Health, Bielefeld University, 33615 Bielefeld, Germany; lennert.griese@uni-bielefeld.de (L.G.); doris.schaeffer@uni-bielefeld.de (D.S.); 2 Department Public Health and Education, Hertie School, 10117 Berlin, Germany

**Keywords:** health information sources, health literacy, focus groups, people with chronic illness, HLS-GER 2, Germany

## Abstract

Background: People with chronic illness are particularly dependent on adequate health literacy (HL), but often report difficulties in accessing, understanding, appraising, and applying health information. To strengthen the HL of people with chronic illness, in-depth knowledge about how they deal with health information is crucial. Methods: To this end, quantitative data from the Second Health Literacy Survey Germany (HLS-GER 2) and qualitative data from seven focus group discussions were used to examine the interest in health information, preferred sources of information as well as experiences and challenges with information management among people with chronic illness. Results: The results show that people with chronic illness have a great interest in health information and use very different sources of health information, preferring personal information from physicians most. The results also point to several challenges in health information management that seem to be influenced by the illness duration as well as by the experiences made with the respective sources. Conclusions: Overall, the study provides important starting points for intervention development for the provision and communication of health-related information, but also to research on health information behavior and HL.

## 1. Introduction

Chronic diseases and enduring health problems constitute a major global challenge. They account for 71% of deaths worldwide and are the main determinant of the morbidity spectrum [1,2]. They are always coupled with a high demand for information, which is not uniform, but changes frequently over the course of illness, and becomes more extensive and multi-layered as the complexity of the medical condition increases [3,4,5,6]. 

For many years, people with chronic illness in Germany faced a lack when searching for information. Access to information was also inadequate. In the meantime, the situation has changed fundamentally. Triggered by digitalization, there is now an overload of information and information opportunities, also referred to in the discussion as ‘information obesity’ [7]. At the same time, the amount of misinformation and disinformation as well as advertising-supported and manipulated information has increased [8,9]. Consequently, new difficulties have arisen and information management—especially information accessing and appraising—has become a more complex and demanding task.

It is therefore not easy to meet the associated requirements, and this demands not only sufficient, easily accessible and comprehensible information, but also adequate health literacy [10]. In the context of chronic illness, health literacy can be understood as the motivation and ability to access, understand, appraise, and apply health-related information to cope with the challenges of living with chronic illness; to actively participate in the treatment, recovery, or preservation of health stability and the decisions necessary to do so; to navigate the healthcare system; and to cooperate constructively with healthcare professionals. Overall, it should aim at achieving an optimal management of the medical condition and the best possible treatment and health care [11,12]. However, available research shows that people with chronic illness often have low health literacy levels and face a host of problems in managing health-related information [13,14,15,16,17]. To comprehend these problems, it is necessary to gain a better understanding of the health information behavior of people with chronic illness, the significance of different information sources, and the experience gained with both. 

Previous research on health information behavior in general has focused on different strands, such as the type and extend of information sought, the information needs and preferred sources as well as the personal and source-related characteristics affecting health information behavior [18,19,20,21]. In this context, especially the significance of individual information sources has been emphasized in the past: both quantitative and qualitative studies show that doctors particularly are the primary source of health information, but also that the Internet has become increasingly important [21,22,23,24,25,26].

In terms of chronic illness, research has shown that people with chronic illness are confronted with many, very different problems for which they need comprehensive information [5,27,28]. Similarly, studies show that information acquisition and management are an important part of coping with chronic illness, for which a lot of time and energy is spent [6,19]. When searching for health information, people with chronic illness use a wide range of information sources for different purposes [29,30,31], including various interpersonal sources, traditional, and new media, which are often used simultaneously [19,32]. Although the previously mentioned preferences for physicians as the most important source of health information have also been confirmed in the context of chronic illness [31,33], it must be assumed that the information behavior in the chronically ill is strongly influenced by several personal and contextual factors [34]. It is likely that health information behavior differs from stage to stage in the illness trajectory [35,36,37]. The same applies to information receptivity or absorption capacity, which also varies and is strongly dependent on the situation [10,37,38]. Moreover, the preferences for information sources and information needs are also strongly influenced by the illness situation (e.g., the progression of the chronic illness and/or therapy), the duration of illness, as well as by the experiences made with the respective sources [33,34,39,40].

However, to date, most of the results on health information behavior available in literature relate to selected diseases, such as cancer or diabetes (e.g., [18,30,41]), using either quantitative or qualitative data. There is a lack of studies, especially for Germany, combining both perspectives to provide insight into the preferences and motives for using different sources of information among people with chronic illness and which shed light on their information management. Therefore, the present article attempts to fill this research gap by analyzing: (1) the interest in health information and motivation for information management among people with chronic illness; (2) the sources used by people with chronic illness; and (3) their experiences with different sources of information and their information management, as well as the challenges they face in this context. 

## 2. Materials and Methods

A mixed-methods approach was chosen to answer these questions. Data from the Second Health Literacy Survey Germany (HLS-GER 2) [42] as well as the results from seven focus group discussions [43] were used and analyzed. The HLS-GER 2 is an extended follow-up survey of the first German Health Literacy Survey (HLS-GER 1) [15] and was conducted within the framework of the international comparative study HLS_19_ of the M-POHL Network of the WHO Europe [44]. The HLS-GER 1 provided initial findings on health information management of the German population and formed the basis for focus group discussions [43], which presented first in-depth data among people with chronic illness. These findings were combined with new data on health information management of the German population provided by the HLS-GER 2 [42]. The aim of this combination was to complement the findings from the quantitative analysis with the experiences and challenges in health information management from the perspective of people with chronic illness to better understand their health information behavior.

The HLS-GER 2 involved a total of 2151 people aged 18 years and older living in Germany who participated in face-to-face interviews (PAPI) between December 2019 and January 2020. For measuring chronic illness, it was asked if respondents have any long-term illness or health problem, which has lasted or is expected to last for 6 months or more. Overall, 1086 of the respondents stated that they were affected by at least one chronic disease [42]. About one-third (30.1%) had one chronic disease, and 69.9% suffered from multiple chronic diseases. The average duration of illness was approx. 13 years (13.20; SD = 11.72). There were slightly more women (53.3%) than men (46.6%) with chronic conditions in the sample. On average, the respondents were 58.67 years old (SD = 16.63) (Table 1).

In addition to health literacy, the HLS-GER 2 also took a closer look at some aspects of health information behavior. Among other things, the questions covered interest in and motivation for dealing with health information, preferred sources of information, and experiences with these sources in understanding information (for more details see [42]). For the present analysis, the HLS-GER 2 findings were used and analyzed specifically for people with chronic illness. One main focus of the analysis is on stratification by duration of illness.

Furthermore, data were analyzed from a total of seven focus group discussions conducted between November 2017 and February 2018 on the perspectives and experiences of people with chronic illness in managing health information [43]. The focus groups were made up of five randomly composed patient groups (individuals with HIV/AIDS, tumor diseases, cardiovascular diseases, chronic pain, and rare chronic diseases). Contact was established through self-help facilities [45]. The members of two other focus group discussions were recruited by a survey institute and were individually assigned to a group. The participants in these focus groups were also chronically ill or were relatives of a chronically ill person (Table 2).

Each focus group consisted of four to nine discussants. A total of 41 people participated in the discussions, which were structured thematically and followed a guideline focusing on four thematic complexes derived from empirical findings on health information management of the German population [15]: (1) Understanding of health literacy; (2) significance and use of different health information sources as well as experiences made with health information management in different sources; (3) challenges in information management during the course of illness; (4) suggestions for improving health information and facilitating information management. The discussions lasted 90–120 min. Each participant consented to the discussions being recorded, transcribed, and anonymized. For the subsequent analysis, the data were sequenced and coded according to the topic. In addition, code trees were created. The codes, which were mainly derived from the data material (in vivo codes) [46], were then arranged in an organizational structure based on a first rough interpretation of the data. In a next step, the respective text segments were matched and then analyzed in detail. At the same time, the classification structure already developed was reviewed and modified where necessary.

## 3. Results

### 3.1. Interest in Health Information

**Figure 1:**
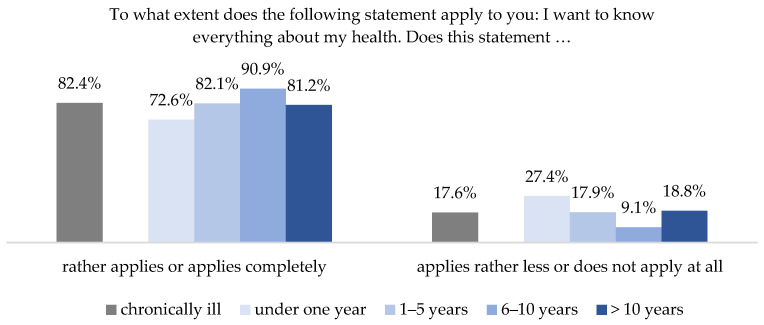
Interest in health information differentiated by duration of chronic disease (HLS-GER 2).

Overall, people with chronic illness are highly interested in information on health and illness. According to the HLS-GER 2, more than 82% of the respondents with chronic conditions agreed with the statement that they wanted to know everything about their health. Only 18% could not identify with this statement [42]. A differentiated analysis according to the duration of chronic illness showed that interest in health information is lower in the first year following diagnosis, at 72.6%, but increases to up to 90.9% as the condition progresses. After having lived with the disease for more than 10 years, interest declines again, but is still higher than in the first year after diagnosis (Figure 1).

This tendency is also reflected by the focus groups, which also reported a greater interest in health information as the duration of illness increased. The participants emphasized that they experienced a crisis at the beginning of their illness and had to deal with the shock of the diagnosis, along with the resulting disruption of their previous reality (see also [47,48]). Therefore, their interest in extensive professional information was limited at this stage.

*“The patient is already ill and must first cope with the disease and is then bombarded with specialist information (.), which has nothing to do with the individual patient.”*

(FG 4)

The last sentence is particularly noteworthy, because it shows that the capacity of people with chronic illness to absorb information at the time of diagnosis is limited. Moreover, the information received appears to be focused on specialist or medical textbook information that does not consider the patient’s individual situation and the psychological and social stress of receiving a diagnosis. 

Only when the shock of the diagnosis has lessened and there is hope of a return to normality, patients begin to take a greater and more active interest in information. At the same time, the desire for further information grows successively with an increase in the duration of the disease:

*“Yes definitely. Dealing (with health information) has become the central focus of my life. Every bit of information and every source is checked over and over.”*

(FG 7)

Moreover, not only how often, but also in which manner people search information seems to change with longer illness duration:

*“I would say it has become more intense, more positive, much more targeted. So not taking in everything anymore, but really only targeted.”*

(FG 2)

The quotes make clear that the management of health-related information is not only gaining in importance and scope, but is gradually becoming an integral part of life because every piece of information found, and every source of information used, is thoroughly and critically examined. The resulting difficulties for information management and especially for accessing and appraising information will be considered in the following.

### 3.2. Health Literacy among People with Chronic Illness

People with chronic illness are particularly dependent on health information and also on adequate health literacy, i.e., the ability to manage health information. However, the data of the HLS-GER 2 study show that almost two thirds (62.3%) of the people with chronic illness have difficulties with information management and thus show low health literacy levels. Especially appraising health information is particularly challenging. Overall, 76.4% of people with chronic illness have difficulties in this area. However, accessing information also poses challenges. More than half (51.8%) of the respondents with chronic illness report difficulties here [42]. This is also reflected in the focus groups, where the assessment of information is also seen as very challenging.

*“So, judging I sometimes find difficult because there is always this opinion and that opinion (...) That’s why it is sometimes really hard to judge what’s good for me and not and what I should do now.”*

(FG 1)

As the quote shows, difficulties arise from the amount of different information, but also the different quality of information causes uncertainty and requires critical judgment.

*“I don’t need to read this page any further, because it is all about selling me something. You have to be very careful.”*

(FG 2)

Similar to the interest in health information being highest at 6–10 years of illness duration, accessing and appraising information is also most difficult at this stage. Overall, 60.8% of the people with a chronic illness lasting 6–10 years have problems finding appropriate information, and 85.1% consider it challenging to appraise the information they find. These values are significantly higher than those for chronically ill people with a shorter duration of illness. Of those who have been chronically ill for less than a year, 53.7% report difficulties in finding and 62.7% in appraising health information. Of those having a chronic disease for 1–5 years, 49.7% consider it challenging to find health information and 76.3% to appraise them.

### 3.3. Preferred Sources of Information

**Figure 2:**
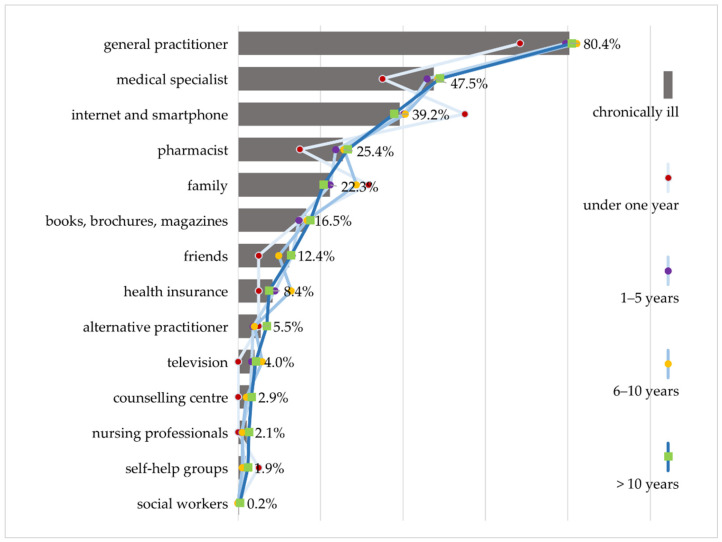
Preferred sources of information differentiated by duration of chronic disease (HLS-GER 2).

When asked which sources of information they prefer, people with chronic illness show a clear preference for information from doctors (Figure 2). For 80.4% of the respondents with chronic illness interviewed in the HLS-GER 2, primary care physicians are the most important source of health information, while just under half (47.5%) prefer medical specialists. The great importance of physicians as a source of information is also expressed in the focus groups. This is explained by the high level of trust placed in physicians. Information from physicians is predominantly regarded as credible, reliable and of high quality.

At 39.2%, the Internet ranks third in the hierarchy of preferred sources of information for people with chronic illness, behind general practitioners and specialists. According to the focus groups, it is mostly used to obtain an initial overview of the symptoms, effects, and treatment options.

*“So, I am very much googling and very often on Wikipedia. If a clinical picture comes up somewhere that affects not only me but also my family (…). When mom has a weird cough, I’m already looking, what could it be?”*

(FG 1)

At the same time, the Internet is used as a means of reassurance:

*“When I was diagnosed, when the doctor told me what I had, I got on the Internet and researched what it meant. She did tell me a few things (...), but then I got more detailed information from the Internet.”*

(FG 5)

This quote shows that the Internet also serves as a supplementary source of information that allows patients to search for more in-depth or reassuring information before and after visiting the doctor. The search for structured and qualified information, as well as the need for emotional support and opportunities for exchange, are cited as further motives for using the Internet. 

Pharmacies also play an important role in providing information. A quarter (25.4%) of people with chronic illness surveyed in the HLS-GER 2 used information provided by pharmacies. Pharmacists are the first point of contact for questions about the patient information leaflet, which more than half of the people with chronic illness surveyed in HLS-GER 2 (58.8%) found difficult to understand [42], as well as for questions about effects, tolerability, and interaction with other medications, and especially for complex medication regimes.

*“If I am prescribed something new, because I receive medication from different doctors, then (...) the pharmacy is my point of contact to find out if the medications are all compatible (...) And I have a really competent pharmacy (...) that checks the medications against each other.”*

(FG 2)

This quote shows that the role of the pharmacy as a hub of information is particularly valued. It bundles information obtained elsewhere and checks prescribed medications for compatibility or adverse side effects.

Approximately one-fifth (22.3%) of people with chronic illness prefer to obtain information from family members. The focus groups show that they pave the way for access to health information and are also important for people with chronic illness in further managing information, since they explain things that have been misunderstood and provide support in understanding information.

*“Yes, then I show it to my sons, and they tell me what it means. I don’t understand everything, and they explain it to me.”*

(FG 2)

The family plays a particularly important role in classifying, assessing, and processing health information, and is perceived overall as a trustworthy and helpful complementary support for information management. 

As with the interest in health information, individual sources of health information are assigned varying degrees of relevance depending on the illness duration (Figure 2). According to the HLS GER 2, doctors and pharmacists become more important the longer the illness lasts. During the first year, especially the Internet and family members seem to be the most important sources of information for people with chronic illness. It can be assumed that emotionally overcoming the acute crisis and the shock of diagnosis is the motivation behind the search for information, which makes detailed specialist information of secondary importance. According to the focus groups, sharing information among the family or searching online for the experiences of people with chronic illness is helpful to better understand their own situation, alleviate fears and overcome the shock of their diagnosis.

### 3.4. Experience in Searching for and Dealing with Health Information

People with chronic illness are usually very experienced in dealing with health information from the sources mentioned here. Four overarching themes emerge that are key in choosing these individual sources. These include *trust* in the source and the perceived competence, the time available and the comprehensibility of the information. If people with chronic illness are dissatisfied with any of these factors, they often continue to search for and use other sources of information.

#### 3.4.1. Trust and Competence 

Trust is a basic prerequisite for the use of certain information sources. Overall, physicians enjoy a high degree of trust, but do not always succeed in providing their patients with the information they desire. In the HLS-GER 2, 29.6% of people with chronic illness report significant difficulty in obtaining the exact information they need from their doctor [42]. Participants in the focus groups confirm this and emphasize how much this undermines their trust in competence because they believe trust is the most important prerequisite for a functioning doctor–patient relationship. People with rare chronic diseases especially, or those with symptoms that are difficult to diagnose, tell of numerous experiences where they received less than satisfactory information. This often leads to annoyance, confusion, uncertainty, and results in serious consequences for using health information sources:

*“You start to search around when you feel uncertain and don’t know what to do and reach a point where you just feel so alone, and that’s when something has to happen. You either begin to look for other doctors or whatever.”*

(FG 7)

Trust usually begins to erode when a patient starts to doubt the competence surrounding information, which is perceived to be unsatisfactory from a user perspective. This often leads to a search for further information, usually on the Internet, or a change of doctor is considered. 

While physicians enjoy a high level of trust as sources of information, much of the information found on the Internet is regarded with skepticism. This skepticism results from the amount of contradictory and interest-driven information available on the Internet, which places high demands on information search and assessment. According to the HLS-GER 2, 83.5% of people with chronic illness consider it difficult or very difficult to assess the trustworthiness and reliability of digital information. A further 63.2% have difficulty in finding the exact information they are searching for on the Internet [42], which is also confirmed by the focus groups.

*“When you search for such and such on the Internet, the first things that always appear are the worst things you could have, and that’s more unsettling than it is reassuring. That’s why it’s better to go to the doctor.”*

(FG 6)

This clearly shows that searching for information on the Internet is not only time-consuming, but also frustrating and unsettling due to the large amount of unreliable and low-quality information.

The family also plays an important role in this context. They are greatly trusted as a source of support and advice when uncertainty and confusion arise related to information management. To some extent, the family also assumes a protective function.

*“My daughter always says: Stay away from the Internet, go to the doctor instead. If you Google, you’ll be dead in six months.”*

(FG 6)

As the quote shows, this also implies that it may not be advisable to use certain information sources. 

The level of trust in individual sources of information fluctuates with an increase in the duration of an illness and the accumulation of experience with managing information and various information sources. Overall, the attitude toward information becomes more critical and the trustworthiness of individual sources of information is questioned more closely as a result. In the first year following their diagnosis, 69.1% of people with chronic illness find it difficult to assess the trustworthiness of digital information, in contrast to 89.5% of respondents with an illness lasting 6–10 years. Focus groups emphasize that this not only applies to digital information, but also to how they behave as patients.

*“And then his (the physician’s) statements need to be checked. And I do check them now but didn’t ten years ago.”*

(FG 5)

As this quote demonstrates, attitudes and actions regarding established and trusted sources of information (in this case doctors) change with an increase in the duration of illness. 

#### 3.4.2. Time 

In addition to trust, the time available to manage information plays an important role. According to the HLS-GER 2, 49.4% of respondents with chronic illness state that obtaining enough consultation time is the most difficult aspect of interaction with their physicians [42].

*“But that’s just chop-chop: waiting three hours for five minutes, then you have a piece of paper in your hand with a medication and then you leave.”*

(FG 2)

To address this, many patients develop targeted strategies to effectively use the narrow timeframe available.

*“They don’t have any time. That’s why I (…) wrote down my questions beforehand, so I knew what to ask. But I still don’t have the feeling that I know everything I should, because they just didn’t take any time with me.”*

(FG 6)

However, these strategies do not always produce the desired results for people with chronic illness. Some have shared their experiences concerning physicians who make ironic comments about their efforts and whose approaches are less than constructive. Actively seeking information is therefore often perceived as negative.

*“And then you’re considered the worst kind of patient if you’ve done your research beforehand! And oh brother, we’ve all gone through that at least once. When you already know a few things and go to the doctor—forget it, not a chance.”*

(FG 7)

Experiences like these encourage people with chronic illness to adopt traditional, passive patient behavior and to forgo active participation, including requesting information. This also corresponds to the results of the HLS-GER 2, in which 34.7% of people with chronic illness assess communicating their personal views or ideas to their physician as (very) difficult. Nearly one-third (32.1%) find it difficult to participate in decisions that affect their own health [42].

According to the focus groups, frequently long waiting periods for an appointment and very brief consultation periods that leave little time for questions or further information limit the opportunity for more participation and co-production. These issues often lead to annoyance, which in turn leads to the search for information elsewhere. 

The focus groups point out that a positive aspect of online searches is that they can take place any time and without an appointment. In addition, such searches are not limited to a certain timeframe and can be carried out until the desired information has been found. However, Internet searches can quickly become very time-consuming, since the information must be filtered out from a large number of search results. Searches also do not usually lead directly to the information sought.

*“But then one page leads to another page and another and there’s more and more information (...) and you continue reading and suddenly there are 1000 tabs open and at the end you’re just confused.”*

(FG 7)

This quote also shows that it is not only the abundance of information that makes searching difficult, but also the fragmentation of information or the lack of user-friendly guidance systems and navigation aids through digital space that causes disorientation.

**Figure 3:**
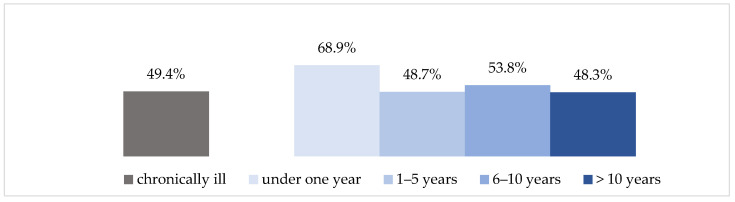
Difficulty in obtaining sufficient consultation time (HLS-GER 2).

Regarding the illness duration, the first year following the diagnosis is also particularly challenging here. As Figure 3 shows, people with chronic illness find it by far the most difficult during this phase to obtain sufficient consultation time with their physician.

#### 3.4.3. Comprehensibility of Information and Communication 

**Figure 4:**
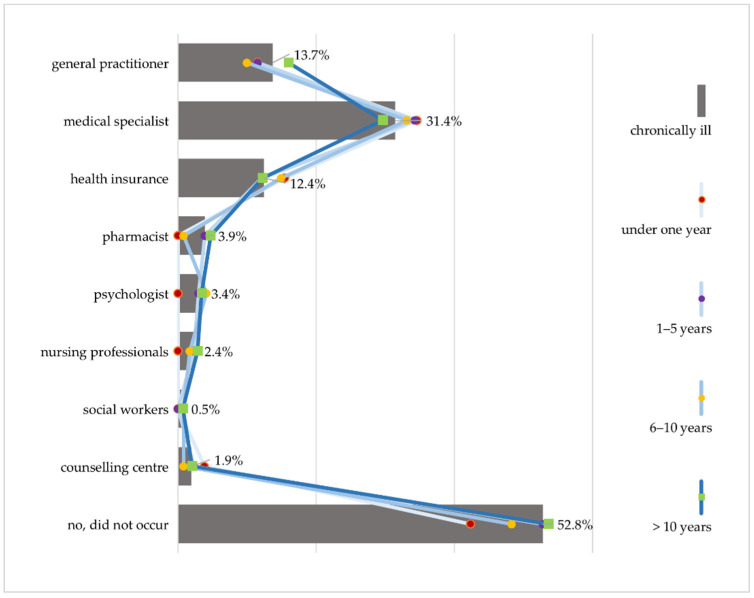
Difficulty in understanding explanations by healthcare providers differentiated by duration of chronic illness (HLS-GER 2).

Communication that is easily comprehensible is another important criterion in choosing the medium of information. However, problems frequently arise here, as well. Almost half of the HLS-GER 2 respondents with chronic illness did not understand explanations given by a health professional at least once in the last 12 months. Difficulties in comprehension occur most frequently in communication with doctors. Overall, 31.4% report problems in understanding explanations by their specialists and 13.7% by their general practitioners (Figure 4). This is in line with the 47.2% of people with chronic illness who assess it as (very) difficult to understand the terms used by their doctors [42].

Focus groups also frequently criticize the communication with physicians. Despite positive developments, focus group participants claim that doctors still express themselves too abstractly and use too many medical terms.

*“I once had a doctor, an orthopedist. The receptionist was there during the examination. The doctor just rattled off something in Latin, went out and then the receptionist said: Okay, I’ll translate for you, you probably didn’t understand anything.”*

(FG 7)

Some even suspect that this mode of communication is intentional to ensure patient compliance.

*“We’re not supposed to understand, that’s why they also use the Latin medical terms. Patients are kept in the dark so they can’t raise any objections or take matters into their own hands, which could be considered contra-productive (...).”*

(FG 3)

Taking the illness duration into consideration shows that difficulties in comprehension tend to decrease over time. While 57.7% report difficulties in understanding during the first year of diagnosis, this proportion is much lower at 46.4% among respondents with an illness of longer than 10 years. The results of the focus groups provide a possible explanation for this.

*“When my doctor or a specialist now throws around a medical term, I immediately say: What does that mean? If I don’t know something, I ask, but there are also people who are too afraid or shy to ask questions.”*

(FG 6)

According to the focus groups, people with chronic illness grow into an active role as patients over time, ask more explicitly for comprehensible information, ask questions if they have not understood something correctly and want to be involved in decisions. At the same time, this is perceived as difficult because it requires a departure from traditional notions of the patient role, which, as previously mentioned, demands a great deal of effort because it is not supported by all physicians. This is the reason why focus groups advocate for more person-centered care, more sensitive communication, and a more perceptive style of interaction. 

## 4. Discussion

The aim of the article was to generate in-depth knowledge about how people with chronic illness deal with information and information management. To this end, quantitative and qualitative data on information use were analyzed to examine interest in health information, preferred sources of information as well as experiences and challenges with information management.

The results show that people with chronic illness have a great interest in health information and are for the most part quite active in searching for and requesting information. However, this appears to be linked to the duration of chronic illness. In the first year after diagnosis, people with chronic illness usually find it difficult to deal with health and illness information, because they have to overcome the shock of the diagnosis and often find themselves in an acute crisis situation [48]. With an increase in the duration of illness, they become more interested in information, ask more specific and in-depth questions, and engage more intensively with illness and health information [48,49]. At the same time, they perceive information management to be more difficult and their health literacy is declining. This is surprising, because it could be assumed that a gain in competence would occur through the accumulation of experience. However, another interpretation is also possible. Precisely because people with chronic illness deal with information more intensively, they might assess the difficulties associated with information management more realistically and more critically—especially in terms of coping with the challenges associated with the abundance of information, such as finding the right information and being able to assess how reliable and trustworthy it is. This is not only critical for health information behavior, but also for disease management and care, as shown by studies on the effects of low health literacy and associated difficulties in information management [42,44]. This must be taken into consideration, as well as that the need for information varies depending on the stage of the disease and that not every time is the right time to provide it. As the results confirm, receptiveness to information is limited immediately following diagnosis and in times of crisis [48], yet information is often provided exactly during such periods. Therefore, it is important to support people with chronic illness through trajectory-oriented information management that takes into account the ever-changing need for new information during the course of the disease while remaining centered on the patient and on equal footing with them [10,11,49]. 

At the same time, the results also show that people with chronic illness consistently use very different sources of information, and prefer their information orally as opposed to in writing. This is often overlooked in the current discussion, which focuses primarily on written information and ways to improve it, and frequently targets a single source of information [50]. However, in our view, it is important to pay more attention to the mix and interplay of sources—especially from an intervention point of view, because according to our results, some sources of information, such as the Internet, serve especially to reassure patients who use it as a form of support. 

The results show that physicians are the most important source of information; a number of other studies have also come to this conclusion [22,23,51]. This corresponds to the high status and social position doctors still enjoy in many countries, especially in Germany [52]. Both of these factors explain why, from the perspective of people with chronic illness, physicians are the first point of contact when seeking health information, and enjoy a high level of trust as a reliable and high-quality source of such information [25,31,34,53]. 

However, our analysis also points to the growing relevance of other sources of information in Germany—most notably the Internet [22,54,55,56]. A few years ago, the Internet was in fourth or fifth place on the list of popular information sources in Germany [15,56,57], but is now in second place after physicians [22,23] as a supplementary source of medical information, and becomes even more important when the trust between doctor and patient begins to erode. This is a curious finding, given the lagging development of digitalization in Germany [58,59]. 

As the focus group discussions specifically suggest, this is due in many cases to misgivings, confusion, and the resulting criticism of the communication and interaction with doctors, especially the lack of consultation time. Other studies confirm this finding [31,37,60]. Compared internationally, the number of physician consultations in Germany is much higher than in other countries, but also significantly shorter, which means that only a limited timeframe for in-depth information is available [61,62]. An adequate consultation period is especially important for people with chronic illness to communicate their wishes and views, and to participate actively and co-productively in their own treatment and care. According to the results of this study, however, a large number of people with chronic illness are unable to do so; this shows that the call for longer consultation periods and structural changes that has become louder over the years is still relevant [63,64]. This also applies to the communication with doctors. It has improved, as the results of this study show, but is relatively insignificant in everyday life, is usually from a purely medical point of view and is often incomprehensible, too complicated in terms of language, and too little geared to the problems and preferences of patients [42,65]. Improving the communication and interaction skills of physicians, as is currently being discussed in Germany [66], is therefore a high priority from the perspective of people with chronic illness.

In addition, as was repeatedly emphasized in the focus group discussions, the shift toward informed and critical patients is often met with rejection on the part of physicians. This often leads to a loss of trust and is the reason why people with chronic illness begin to ‘shop around’ and consult other sources of information—whether for reassurance or in the search for reliable information [49,60,67]. The Internet, but also the family, assume particular importance, especially during the initial stage of an illness. As other studies also show [31,67], both are a relevant source, especially in the search for emotional support and peer-to-peer exchanges. This underscores how important it is to improve the competences and skills of doctors.

However, using digital information is not easy. Finding the right health information among so much contradictory information on the Internet is difficult and time-consuming, as is distinguishing reliable information from the abundance of false information available. This is confirmed by the recent data on the population’s digital health literacy [42,44,68,69,70,71]. Therefore, bundling tailored, evidence-based, comprehensible information on the Internet and creating information-related guidance systems is especially important for people with chronic illness and their individual information needs that constantly change over time. Initial efforts to this end, such as the creation of information portals, can already be observed in several countries such as Germany (www.gesund.bund.de, accessed on 13 December 2021), England (www.nhs.uk, accessed on 13 December 2021), Denmark (www.sundhed.dk, accessed on 13 December 2021) or Australia (www.healthdirect.gov.au, accessed on 13 December 2021). However, these services are usually not yet tailored to the specific needs of people with chronic illness and are not automatically available to users, but must be accessed independently. Improving this by shifting pull into push could give people with chronic illness the ability to face the challenges of personal responsibility and self-management that is expected of them at each stage of their disease to cope with their illness, as well as the difficulties that arise in managing the related information. 

## 5. Limitations

There is a lack of findings that shed light on information management and the experience of using different health information sources from the perspective of people with chronic illness, especially in Germany. By using a mixed-methods approach, findings are now available based on an extensive database. However, there are also limitations; although the two surveys are closely linked, as they belong to a series of studies that build on each other starting in 2016 with the first German Health Literacy Survey (HLS-GER 1) [15], it must be taken into account that they are still two independent samples. In addition, the present study examines the importance of the duration of illness, but not the age of participants in regard to information use and information management. However, it can be assumed that age plays an important role, especially for the preference for digital information sources. A similar limitation must be made regarding other socio-demographic and socio-economic determinants that have already been found to be significant for information behavior as well as for health literacy in people with chronic illness [16,18]. This should also be taken into account when interpreting the results and should be examined in more detail in future analyses.

## 6. Conclusions

Overall, the results of the study provide important starting points for intervention development and illustrate that too little attention has been paid to the perspective of people with chronic illness. This applies not only to the provision and communication of health-related information but also to research on health information behavior and health literacy. More attention should be paid to the patient view in both of these cases. In order to support people with chronic illness in their health information management, the following starting points can be summarized from the previous results:

- Establish a trajectory-oriented information management that takes into account the ever-changing needs of people with chronic illness.
- Consider the mix of different information sources and, in addition to improving written information, pay particular attention to oral information and communication with health professionals.
- In doing so, foster the necessary structural changes and anchor skills and competencies required for information provision in the education and training of health care professionals.
- Establish special guidance systems and navigation aids for people with chronic illness that make it easier to find and use health information along the entire illness trajectory and thereby increasing health literacy.

## Figures and Tables

**Table 1:** Sample characteristics HLS-GER 2 (n = 1086) ^1^.

Variable	Proportion/Mean (SD)	N
**Age** [min, max: 18–92]	58.67 (16.63)	**1080**
18–29 years	7.3	79
30–45 years	15.3	165
46–64 years	36.1	390
65 years and older	41.3	446
**Illness duration** [min, max: 0–82]	13.20 (11.72)	**1066**
less than one year	2.4	26
1–5 years	28.6	305
6–10 years	11.3	121
>10 years	57.6	614
**Number of chronic diseases** [min, max: 1–12]		**1086**
one	30.1	327
more than one	69.9	759
**Gender**		**1084**
male	46.7	506
female	53.3	578

^1^ Weighted sample based on the population structure of the German Microcensus 2018 adjusting for gender, age, population density, state, and education.

**Table 2:** Characteristics of the focus group participants (n = 41).

Variable	Proportion/Mean (SD)	N
**Focus group participants**		**41**
FG1 AIDS		4
FG2 chronic pain		7
FG3 colon cancer		5
FG4 chronic ischemic heart disease		4
FG5 rare chronic illnesses		4
FG6 mixed group by survey institute		8
FG7 mixed group by survey institute		9
**Age** [min, max: 27–83]	58.34 (14.83)	**38**
**Gender**		**41**
male	56.1	23
female	43.9	18

## Data Availability

All data relevant to the study are included in the article. For further questions regarding data availability, please contact the authors.

## References

[1] World Health Organization (2018). Noncommunicable Diseases Country Profiles.

[2] Eurostat Personen mit Einem Lang Andauernden Gesundheitsproblem, Nach Geschlecht, Alter und Erwerbsstatus: Europäische Gesundheitsstatistiken. https://ec.europa.eu/eurostat/databrowser/view/hlth_silc_04/default/table?lang=de.

[3] Hyman R.B., Corbin J. (2001). Chronic Illness.

[4] Schaeffer D. (2009). Bewältigung Chronischer Erkrankungen im Lebenslauf.

[5] Corbin J.M., Strauss A.L., Hildenbrand A. (2010). Weiterleben Lernen: Verlauf und Bewältigung Chronischer Krankheit.

[6] Schaeffer D., Haslbeck J., Richter M., Hurrelmann K. (2016). Bewältigung Chronischer Krankheit. Soziologie von Gesundheit und Krankheit.

[7] Whitworth A. (2009). Information Obesity.

[8] Okan O., Bollweg T.M., Berens E.-M., Hurrelmann K., Bauer U., Schaeffer D. (2020). Coronavirus-Related Health Literacy: A Cross-Sectional Study in Adults during the COVID-19 Infodemic in Germany. Int. J. Environ. Res. Public Health.

[9] World Health Organization (2020). Infodemic management: A key component of the COVID-19 global response. Wkly. Epidemiol. Rec..

[10] Schaeffer D., Schaeffer D., Pelikan J.M. (2017). Chronische Krankheit und Health Literacy. Health Literacy: Forschungsstand und Perspektiven.

[11] Schaeffer D., Schmidt-Kaehler S., Dierks M.-L., Ewers M., Vogt D. (2019). Strategiepapier #2 zu den Empfehlungen des Nationalen Aktionsplans. Gesundheitskompetenz in die Versorgung von Menschen mit Chronischer Erkrankung Integrieren.

[12] Sørensen K., van den Broucke S., Fullam J., Doyle G., Pelikan J., Slonska Z., Brand H. (2012). Health literacy and public health: A systematic review and integration of definitions and models. BMC Public Health.

[13] Rademakers J., Heijmans M. (2018). Beyond Reading and Understanding: Health Literacy as the Capacity to Act. Int. J. Environ. Res. Public Health.

[14] Rowlands G., Protheroe P., Saboga-Nunes L., van den Broucke S., Levin-Zamir D., Okan O., Okan O., Bauer U., Levin-Zamir D., Pinheiro P., Sørensen K. (2019). Health literacy and chronic conditions: A life course perspective. International Handbook of Health Literacy. Research, Practice and Policy across the Life-Span.

[15] Schaeffer D., Vogt D., Berens E.M., Hurrelmann K. (2016). Gesundheitskompetenz der Bevölkerung in Deutschland—Ergebnisbericht.

[16] Schaeffer D., Griese L., Berens E.-M. (2020). Gesundheitskompetenz von Menschen mit Chronischer Erkrankung in Deutschland. Gesundheitswesen.

[17] Sørensen K., Pelikan J.M., Röthlin F., Ganahl K., Slonska Z., Doyle G., Fullam J., Kondilis B., Agrafiotis D., Uiters E. (2015). Health literacy in Europe: Comparative results of the European health literacy survey (HLS-EU). Eur. J. Public Health.

[18] Mirzaei A., Aslani P., Luca E.J., Schneider C.R. (2021). Predictors of Health Information-Seeking Behavior: Systematic Literature Review and Network Analysis. J. Med. Internet Res..

[19] Lambert S.D., Loiselle C.G. (2007). Health information seeking behavior. Qual. Health Res..

[20] Clarke M.A., Moore J.L., Steege L.M., Koopman R.J., Belden J.L., Canfield S.M., Meadows S.E., Elliott S.G., Kim M.S. (2016). Health information needs, sources, and barriers of primary care patients to achieve patient-centered care: A literature review. J. Health Inform..

[21] Ramsey I., Corsini N., Peters M.D.J., Eckert M. (2017). A rapid review of consumer health information needs and preferences. Patient Educ. Couns..

[22] Baumann E., Czerwinski F., Rosset M., Seelig M., Suhr R. (2020). Wie informieren sich die Menschen in Deutschland zum Thema Gesundheit? Erkenntnisse aus der ersten Welle von HINTS Germany. Bundesgesundheitsblatt Gesundh. Gesundh..

[23] Hurrelmann K., Klinger J., Schaeffer D. (2020). Gesundheitskompetenz der Bevölkerung in Deutschland: Vergleich der Erhebungen 2014 und 2020.

[24] Zimmerman M.S., Shaw G. (2020). Health information seeking behaviour: A concept analysis. Health Inf. Libr. J..

[25] Schaeffer D., Dierks M.L., Schaeffer D., Schmidt-Kaehler S. (2012). Patientenberatung in Deutschland. Lehrbuch Patientenberatung.

[26] Jacobs W., Amuta A.O., Jeon K.C. (2017). Health information seeking in the digital age: An analysis of health information seeking behavior among US adults. Cogent Soc. Sci..

[27] Corbin J., Strauss A. (1988). Unending Work and Care: Managing Chronic Illness at Home.

[28] Haslbeck J. (2010). Medikamente und Chronische Krankheit. Selbstmanagementerfordernisse im Krankheitsverlauf aus Sicht der Erkrankten.

[29] Engqvist Boman L., Sandelin K., Wengström Y., Silén C. (2017). Patients’ learning and understanding during their breast cancer trajectory. Patient Educ. Couns..

[30] Longo D.R., Ge B., Radina M.E., Greiner A., Williams C.D., Longo G.S., Mouzon D.M., Natale-Pereira A., Salas-Lopez D. (2009). Understanding breast-cancer patients’ perceptions: Health information-seeking behaviour and passive information receipt. J. Healthc. Commun..

[31] Todd L., Hoffman-Goetz L. (2011). A qualitative study of cancer information seeking among English-as-a-second-Language older Chinese immigrant women to canada: Sources, barriers, and strategies. J. Cancer Educ..

[32] Nagler R.H., Gray S.W., Romantan A., Kelly B.J., DeMichele A., Armstrong K., Schwartz J.S., Hornik R.C. (2010). Differences in information seeking among breast, prostate, and colorectal cancer patients: Results from a population-based survey. Patient Educ. Couns..

[33] Kalantzi S., Kostagiolas P., Kechagias G., Niakas D., Makrilakis K. (2015). Information seeking behavior of patients with diabetes mellitus: A cross-sectional study in an outpatient clinic of a university-affiliated hospital in Athens, Greece. BMC Res. Notes.

[34] O’Leary K.A., Estabrooks C.A., Olson K., Cumming C. (2007). Information acquisition for women facing surgical treatment for breast cancer: Influencing factors and selected outcomes. Patient Educ. Couns..

[35] Schaeffer D., Moers M., Schaffer D., Wingenfeld K. (2014). Bewältigung chronischer Krankheiten—Herausforderungen für die Pflege. Handbuch Pflegewissenschaft.

[36] Chen A.T. (2016). The Relationship between Health Management and Information Behavior over Time: A Study of the Illness Journeys of People Living with Fibromyalgia. J. Med. Internet Res..

[37] Hambrock U. (2018). Die Suche nach Gesundheitsinformationen: Patientenperspektiven und Marktüberblick.

[38] Baumann E., Hastall M.R., Hurrelmann K., Baumann E. (2014). Nutzung von Gesundheitsinformationen. Handbuch Gesundheitskommunikation.

[39] Zare-Farashbandi F., Lalazaryan A., Rahimi A., Hasssanzadeh A. (2016). The Effect of Contextual Factors on Health Information–Seeking Behavior of Isfahan Diabetic Patients. J. Hosp. Librariansh..

[40] Lui C.-W., Col J.R., Donald M., Dower J., Boyle F.M. (2015). Health and social correlates of Internet use for diabetes information: Findings from Australia’s Living with Diabetes Study. Aust. J. Prim. Health.

[41] Kuske S., Schiereck T., Grobosch S., Paduch A., Droste S., Halbach S., Icks A. (2017). Diabetes-related information-seeking behaviour: A systematic review. Syst. Rev..

[42] Schaeffer D., Berens E.-M., Gille S., Griese L., Klinger J., de Sombre S., Vogt D., Hurrelmann K. (2021). Gesundheitskompetenz der Bevölkerung in Deutschland vor und während der Corona Pandemie: Ergebnisse des HLS-GER 2.

[43] Schaeffer D., Vogt D., Gille S. (2019). Gesundheitskompetenz-Perspektive und Erfahrungen von Menschen mit chronischer Erkrankung.

[44] The HLS19 Consortium of the WHO Action Network M-POHL (2021). International Report on the Methodology, Results, and Recommendations of the European Health Literacy Population Survey 2019–2021 (HLS19) of M-POHL.

[45] Dierks M.-L., Kofahl C. (2019). Die Rolle der gemeinschaftlichen Selbsthilfe in der Weiterentwicklung der Gesundheitskompetenz der Bevölkerung. Bundesgesundheitsblatt Gesundh. Gesundh..

[46] Flick U. (2007). Qualitative Sozialforschung: Eine Einführung.

[47] Bury M. (1982). Chronic illness as biographical disruption. Sociol. Health Illn..

[48] Schaeffer D., Moers M., Schaeffer D. (2009). Abschied von der Patientenrolle? Bewältigungshandeln im Verlauf chronischer Krankheit. Bewältigung chronischer Krankheit im Lebenslauf.

[49] Newton P., Asimakopoulou K., Scambler S. (2012). Information seeking and use amongst people living with type 2 diabetes: An information continuum. Int. J. Health Promot. Educ..

[50] Albrecht M., Mühlhauser I., Steckelberg A., Hurrelmann K., Baumann E. (2014). Evidenzbasierte Gesundheitsinformation. Handbuch Gesundheitskommunikation.

[51] Oedekoven M., Herrmann W.J., Ernsting C., Schnitzer S., Kanzler M., Kuhlmey A., Gellert P. (2019). Patients’ health literacy in relation to the preference for a general practitioner as the source of health information. BMC Fam. Pract..

[52] Ebner C., Rohrbach-Schmidt D. (2019). Berufliches Ansehen in Deutschland für die Klassifikation der Berufe 2010: Beschreibung der Methodischen Vorgehensweise, erste Deskriptive Ergebnisse und Güte der Messung.

[53] Stiftung Gesundheitswissen (2020). Statussymbol Gesundheit. Wie sich der soziale Status auf Prävention und Gesundheit Auswirken Kann: Gesundheitsbericht 2020 der Stiftung Gesundheitswissen.

[54] Link E., Baumann E. (2020). Nutzung von Gesundheitsinformationen im Internet: Personenbezogene und motivationale Einflussfaktoren. Bundesgesundheitsblatt Gesundh. Gesundh..

[55] Robert Koch-Institut (2019). Kommunikation und Information im Gesundheitswesen aus Sicht der Bevölkerung. Patientensicherheit und informierte Entscheidung (KomPaS): Sachbericht.

[56] Marstedt G. (2018). Das Internet: Auch Ihr Ratgeber für Gesundheitsfragen? Bevölkerungsumfrage zur Suche von Gesundheitsinformationen im Internet und zur Reaktion der Ärzte.

[57] Baumann E., Czerwinski F., Böcken J., Braun B., Meierjürgen R. (2015). Erst mal Doktor Google fragen? Nutzung Neuer Medien zur Information und zum Austausch über Gesundheitsthemen. Gesundheitsmonitor 2015: Bürgerorientierung im Gesundheitswesen.

[58] Thiel R., Deimel L., Schmidtmann D., Piesche K., Hüsing T., Rennoch J., Stroetmann V., Stroetmann K. (2018). #SmartHealthSystems: Digitalisierungsstrategien im Internationalen Vergleich.

[59] Schmidt-Kaehler S., Dadaczynski K., Gille S., Okan O., Schellinger A., Weigand M., Schaeffer D. (2021). Gesundheitskompetenz: Deutschland in der digitalen Aufholjagd Einführung technologischer Innovationen greift zu kurz. Gesundheitswesen.

[60] Lee K., Hoti K., Hughes J.D., Emmerton L. (2014). Dr Google and the consumer: A qualitative study exploring the navigational needs and online health information-seeking behaviors of consumers with chronic health conditions. J. Med. Internet Res..

[61] Irving G., Neves A.L., Dambha-Miller H., Oishi A., Tagashira H., Verho A., Holden J. (2017). International variations in primary care physician consultation time: A systematic review of 67 countries. BMJ Open.

[62] OECD (2019). Health at a Glance 2019.

[63] Sachverständigenrat zur Begutachtung der Entwicklung im Gesundheitswesen (2009). Koordination und Integration-Gesundheitsversorgung in einer Gesellschaft des Längeren Lebens. Sondergutachten 2009.

[64] Cartwright J., Magee J. (2006). Information for People Living with Conditions that Affect their Appearance: Report I. The Views and Experiences of Patients and the Health Professionals Involved in Their Care—A Qualitative Study.

[65] Hannawa A.F., Rothenfluh F.B., Hurrelmann K., Baumann E. (2014). Arzt-Patient-Interaktion. Handbuch Gesundheitskommunikation.

[66] Hinding B., Brünahl C.A., Buggenhagen H., Gronewold N., Hollinderbäumer A., Reschke K., Schultz J.-H., Jünger J. (2021). Pilot implementation of the national longitudinal communication curriculum: Experiences from four German faculties. GMS J. Med. Educ..

[67] Ayers S.L., Kronenfeld J.J. (2007). Chronic illness and health-seeking information on the Internet. Health.

[68] Griebler R., Straßmayr C., Mikšová D., Link T., Nowak P. (2021). die Arbeitsgruppe Gesundheitskompetenz-Messung der ÖPGK. Gesundheitskompetenz in Österreich: Ergebnisse der Österreichischen Gesundheitskompetenzerhebung HLS19-AT.

[69] De Gani S.M., Jaks R., Bieri U., Kocher J.P. (2021). Health Literacy Survey Schweiz 2019–2021: Schlussbericht im Auftrag des Bundesamtes für Gesundheit BAG.

[70] Shiferaw K.B., Tilahun B.C., Endehabtu B.F., Gullslett M.K., Mengiste S.A. (2020). E-health literacy and associated factors among chronic patients in a low-income country: A cross-sectional survey. BMC Med. Inform. Decis. Mak..

[71] Schaeffer D., Gille S., Berens E.-M., Griese L., Klinger J., Vogt D., Hurrelmann K. (2021). Digitale Gesundheitskompetenz der Bevölkerung in Deutschland: Ergebnisse des HLS-GER 2. Gesundheitswesen.

